# Cobalamin-Independent Methionine Synthase (MetE): A Face-to-Face Double Barrel That Evolved by Gene Duplication

**DOI:** 10.1371/journal.pbio.0030031

**Published:** 2004-12-28

**Authors:** Robert Pejchal, Martha L Ludwig

**Affiliations:** **1**Department of Biological Chemistry, University of MichiganAnn Arbor, MichiganUnited States of America; **2**Biophysics Research Division, University of MichiganAnn Arbor, MichiganUnited States of America; University of California at San FranciscoUnited States of America

## Abstract

Cobalamin-independent methionine synthase (MetE) catalyzes the transfer of a methyl group from methyltetrahydrofolate to L-homocysteine (Hcy) without using an intermediate methyl carrier. Although MetE displays no detectable sequence homology with cobalamin-dependent methionine synthase (MetH), both enzymes require zinc for activation and binding of Hcy. Crystallographic analyses of MetE from T. maritima reveal an unusual dual-barrel structure in which the active site lies between the tops of the two (βα)_8_ barrels. The fold of the N-terminal barrel confirms that it has evolved from the C-terminal polypeptide by gene duplication; comparisons of the barrels provide an intriguing example of homologous domain evolution in which binding sites are obliterated. The C-terminal barrel incorporates the zinc ion that binds and activates Hcy. The zinc-binding site in MetE is distinguished from the (Cys)_3_Zn site in the related enzymes, MetH and betaine–homocysteine methyltransferase, by its position in the barrel and by the metal ligands, which are histidine, cysteine, glutamate, and cysteine in the resting form of MetE. Hcy associates at the face of the metal opposite glutamate, which moves away from the zinc in the binary E·Hcy complex. The folate substrate is not intimately associated with the N-terminal barrel; instead, elements from both barrels contribute binding determinants in a binary complex in which the folate substrate is incorrectly oriented for methyl transfer. Atypical locations of the Hcy and folate sites in the C-terminal barrel presumably permit direct interaction of the substrates in a ternary complex. Structures of the binary substrate complexes imply that rearrangement of folate, perhaps accompanied by domain rearrangement, must occur before formation of a ternary complex that is competent for methyl transfer.

## Abstract 2

By solving the structure of MetE, the authors have shed light on how the chemically difficult transfer of a methyl group from methyltetrahydrofolate to homocysteine can occur

## Introduction

Methionine synthases catalyze the transfer of a methyl group from N5-methyl-5,6,7,8-tetrahydrofolate (CH_3_-H_4_folate) to L-homocysteine (Hcy), the terminal step in the biosynthesis of methionine. Two apparently unrelated families of proteins catalyze this reaction: cobalamin-dependent methionine synthase (MetH; EC 2.1.1.13) and cobalamin-independent methionine synthase (MetE; 5-methyltetrahydropteroyltriglutamate–homocysteine methyltransferase; EC 2.1.1.14) Organisms that synthesize or transport B_12_ encode the cobalamin-dependent enzyme whereas organisms that cannot obtain B_12_ encode only the cobalamin-independent enzyme. Escherichia coli and many other species of bacteria express both enzymes, but mammals utilize only cobalamin-dependent methionine synthase while plants and yeasts utilize only the cobalamin-independent enzyme.

MetH and MetE both face the same mechanistic challenge. They must catalyze the transfer of a very poor leaving group from the tertiary amine, CH_3_-H_4_folate, to a relatively poor nucleophile, the sulfur of Hcy. MetH facilitates this transfer by using cobalamin as an intermediate methyl carrier [1]. Cobalamin accepts a methyl group from CH_3_-H_4_folate at one active site and donates it to Hcy at a second site [2]. In contrast, MetE appears to catalyze the direct transfer of the methyl group from CH_3_-H_4_folate to Hcy [3]. This latter strategy seems to offer a less satisfactory answer to the mechanistic problems: measured k_cat_ values for MetE are smaller than those for MetH by a factor of approximately 50–100.

MetE and MetH both activate Hcy by binding the thiolate form of the substrate to Zn^+2^ [4]. A similar strategy for alkylation of thiol groups is employed in protein farnesyltransferase [5], geranylgeranyltransferase [6], methanol:CoM methyltransferase (MtaA) [7], the E. coli DNA repair Ada protein [8], and betaine–Hcy methyltransferase (BHMT) [9]. However, the sets of zinc ligands and the structures that house the zinc-binding sites are not conserved within this functional family. In particular, the metal ligands and their positions in the sequence are not the same in MetH and MetE. Three cysteines bind the essential zinc in MetH; the first cysteine ligand resides at the end of strand 6 of a (βα)_8_ barrel, and the remaining vicinal cysteine ligands follow strand 8. A histidine and two cysteines have been identified as metal ligands in E. coli MetE by a combination of mutagenesis experiments [10,11] and extended X-ray absorption fine structure (EXAFS) measurements [12]. The relative positions of these residues in the sequence led to the prediction that in a (βα)_8_ MetE barrel the histidine and cysteine ligands would reside at the ends of strands 5 and 8 [4].

In contrast, the sequences of MetE enzymes give few if any clues to the strategy for binding and activation of folate by MetE. Thus, the mode of folate binding is a key question to be addressed by structure analysis. In both MetE and MetH, activation of the leaving group is thought to involve the protonation of CH_3_-H_4_folate in a ternary complex, E·Hcy·CH_3_-H_4_folate in MetE, or E·cob(I)alamin·CH_3_-H_4_folate in MetH [4]. However, the residues that may facilitate protonation have not been identified for either enzyme.

**Figure 1:**
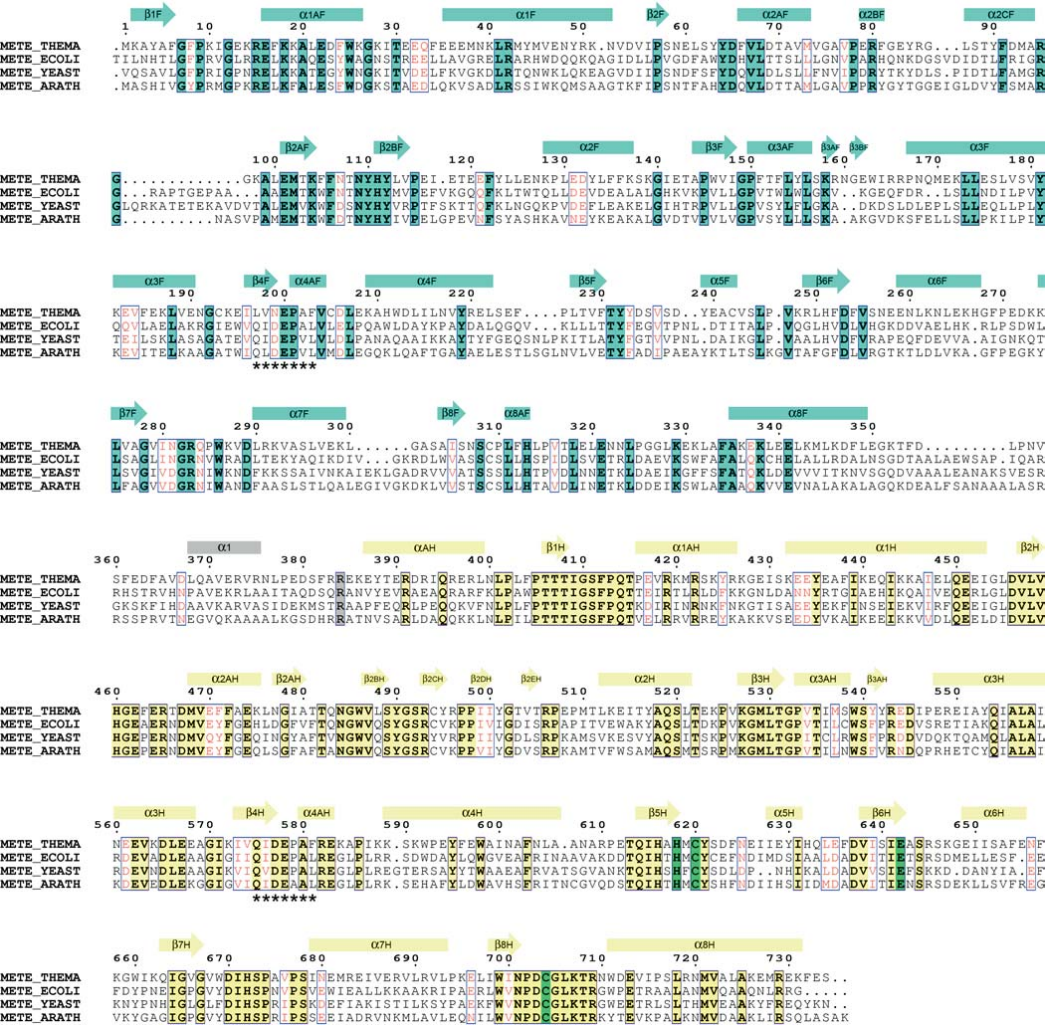
Multiple Alignment of MetE from T. maritima (METE_THEMA), E. coli (METE_ECOLI), Saccharomyces cerevisiae (METE_YEAST), and A. thaliana (METE_ARATH) Conservation in the N-terminal domain is indicated in aqua while conservation in the C-terminal domain is shown in yellow. Zinc ligands His618, Cys620, Glu642, and Cys704 are highlighted in green. The conserved repeat at β4 is marked by asterisks. Main barrel elements are designated β(1–8)F and α(1–8)F and β(1–8)H and α(1–8)H for the N- and C-terminal barrels, respectively. Extension elements are labeled alphabetically and numbered based on the β strand that they follow. For example, α1AF follows β1F and precedes α1F.

MetE appears to have evolved through gene duplication of a sequence encoding a domain of approximately 340 residues that binds and activates Hcy. Within the family of MetE enzymes (Figure 1), the N- and C-terminal halves exhibit significant sequence homology. The C-terminal half is more highly conserved than the N-terminal half and has homologs in archae and elsewhere. Among these thiol methyltransferases are several enzymes that are approximately half the size of MetE and utilize corrinoid proteins, rather than folates, as methyl donors. Taken together, these observations suggested that the MetE gene arose as the result of a primordial gene duplication event followed by loss of zinc- and Hcy-binding determinants from the duplicated sequence [11]. If this hypothesis is correct, the two halves of the MetE sequence should display structural homology, and the N-terminal domain should be more closely related to the C-terminal domain than to any other protein in the database.

To determine how MetE has assembled an active site for catalysis of direct methyl transfer from CH_3_-H_4_folate to Hcy, we have solved the crystal structure of Thermotoga maritima MetE at 2.0 Å resolution, along with structures of the binary substrate complexes with Hcy and folate. Difficulties in crystallization of the E. coli enzyme were circumvented by analyzing the MetE from T. maritima. This thermophilic bacterium encodes orthologs of E. coli MetH and E. coli MetE. T. maritima MetE (TM1286) is 41% identical to the E. coli enzyme and is only 19 residues shorter than E. coli MetE (Figure 1), making it an excellent prototype for the MetE family. MetE comprises two (βα)_8_ barrels. To our knowledge, it is the first example of a dual-(βα)_8_ barrel enzyme in which the active site is located between barrels arranged in a head-to-head orientation. MetE also provides a rare example of a catalytic zinc site in which four residues serve as metal ligands. Repetition of features within the structure supports the idea that MetE evolved through gene duplication of a primordial zinc/Hcy (βα)_8_ barrel.

## Results

### Description of the Fold and Its Evolution

**Figure 2:**
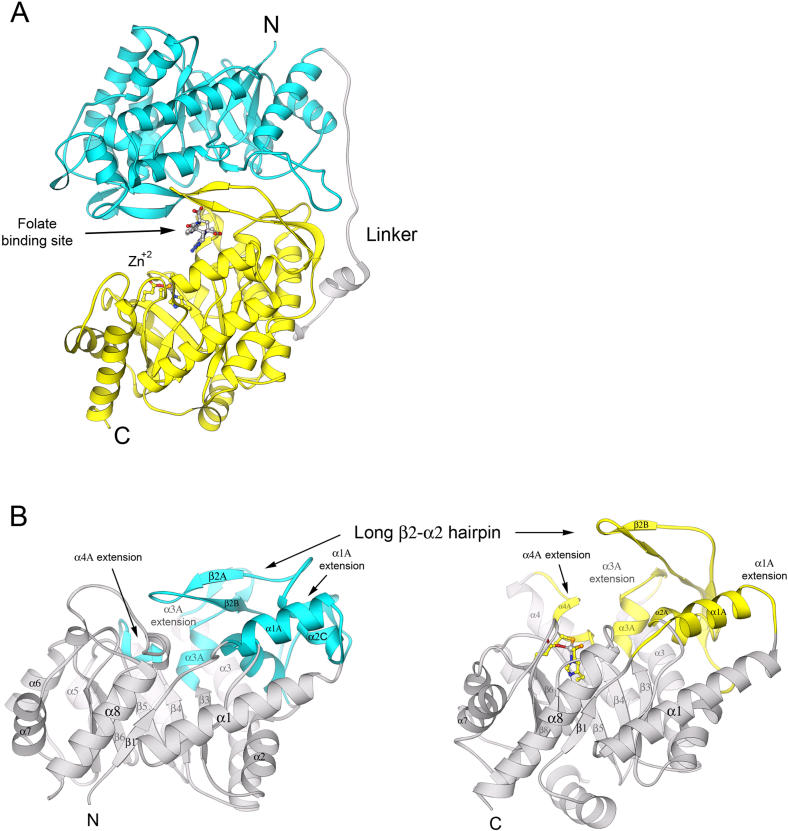
The Fold of MetE and Similarities between the Two Barrels (A) MetE folds into two (βα)_8_ barrels. The N-terminal barrel (aqua) is joined to the C-terminal barrel (yellow) by a 35-residue inter-domain linker (gray) that spans 65 Å. Except for the α1 helix (at the right), the linker residues are in extended conformations. This view is along the approximate 2-fold axis that relates the two barrels. The drawing is based on coordinates for zinc-replete MetE in complex with CH_3_-H_4_folate (Table 1). The zinc ligands, zinc, and CH_3_-H_4_folate are shown in ball-and-stick representation. This figure and all subsequent figures were prepared using RIBBONS [43]. See Figure 1 for the nomenclature used to describe secondary structures. (B) A side-by-side view of the barrels of MetE, arranged to show the similarities of the β–α loop extensions. Major extensions that follow the first four β strands of the barrels are shown in cyan and gold for the N-terminal and C-terminal barrels, respectively. The drawing is based on coordinates for the zinc-replete binary complex with folate (not shown). The active site is located in the C-terminal barrel (on the right) between the extra-barrel β hairpin of the β2–α2 loop and the C-termini of the barrel strands. Zinc is gray and the zinc ligands, His618, Cys620, Glu642, and Cys704, are shown in ball-and-stick mode.

We have determined the structures of several forms of MetE from T. maritima (Table 1), including the zinc-replete binary substrate complexes with folate (the substrate in these experiments was N5-methyl-5,6,7,8-tetrahydropteroyl-(tri)-γ-L -glutamate [CH_3_-H_4_PteGlu_3_]) at 2.59 Å resolution and with Hcy at 2.20 Å resolution. The two (βα)_8_ barrels are formed by residues 1–351 (folate barrel) and 387–734 (Hcy barrel) and joined by an extended inter-domain linker (Figure 2A). An unusual feature is that many of the binding determinants for folate actually reside in the C-terminal barrel that binds Hcy.

**Table 1:**
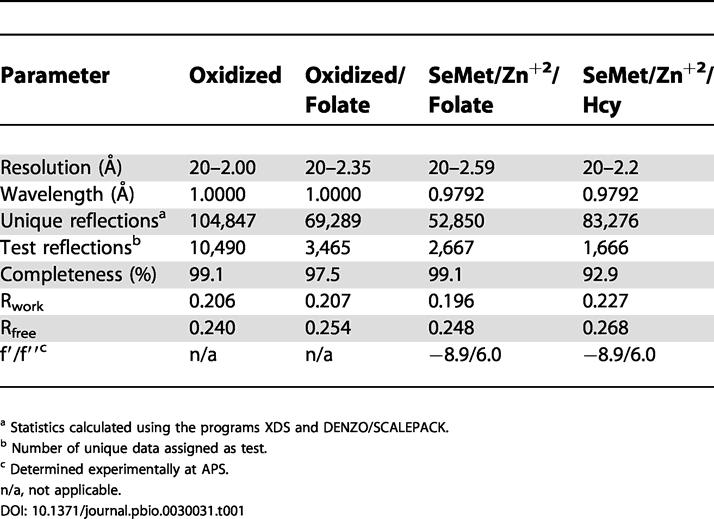
Data Collection and Refinement Statistics for TM1286-HIS

^a^ Statistics calculated using the programs XDS and DENZO/SCALEPACK

^b^ Number of unique data assigned as test

^c^ Determined experimentally at APS

n/a, not applicable

In (βα)_8_ (triose phosphate isomerase [TIM]) barrel enzymes, the active site is usually located near the C-termini of the inner barrel strands, with catalytic residues contributed by the β–α segments (loops) that join these strands to the outer helices. These barrels are topologically polar with their “tops” decorated by insertions that extend the β–α loop segments. MetE is the first example of a dual-(βα)_8_ barrel in which the decorated tops of the two barrels face each other to form a single active site that lies between the domains. A deep cleft between the barrels permits entry of the substrates (Figure 2A). As a result of the arrangement of the barrel domains, residues 352–386 of the inter-barrel linker must span approximately 65 Å to connect the bottoms of the two barrels (Figure 2A).

The N- and C-terminal barrels share a number of strikingly similar features that provide structural evidence for gene duplication. Both barrels incorporate long extensions in the first four β–α loops but, with the exception of α8AF (Figure 2B), lack insertions in the last four loops. A pseudo 2-fold axis superimposes these similar extensions (Figure 2). The helical extension at the start of β1–α1, α1AH, augments the side of the C-terminal barrel and is repeated in the N-terminal barrel. The β2–α2 loops that appear in both barrels are the longest extensions in the structure, and we refer to them as the “long hairpin loops.” In the C-terminal barrel that binds Hcy and Zn^+2^, the long β2–α2 loop begins with helix α2AH and then forms an antiparallel excursion that harbors a number of conserved residues, some of which are involved in binding folate. The β3–α3 loops both include a short helix, α3A, that carries folate-binding determinants in the Hcy (C-terminal) barrel. The β4–α4A segments of both barrels incorporate a conserved sequence, identified by asterisks in Figure 1. In E. coli MetE, this sequence, Gln-Ile-Asp-Glu-Pro-Ala, is identical in both barrels.

Despite the pseudosymmetry that relates the β–α loops of the two barrels, there are significant differences in the sequences and conformations of these connecting loops that distinguish the functional roles of the two barrels. The major binding determinants for the folate substrate lie primarily in the second, third, and fourth β–α loops of the C-terminal barrel. The equivalent binding site in the duplicated N-terminal barrel has been obliterated. Although the long β2–α2 hairpin of the N-terminal barrel resembles the corresponding hairpin of the C-terminal (Hcy) barrel, the potential binding groove for the folate tail is closed by extensive hydrophobic contacts between α1AF, α1F, and α2CF and by interaction with the long hairpin of the C-terminal barrel. The positioning the long β2 hairpin significantly closer to the barrel top than in the C-terminal barrel occludes the pterin-binding site (Figure 2B).

Several other adaptations enhance the barrel–barrel interface and appear to influence the relative orientations of the barrels. The β8–α8 loop containing helix α8AF is 16 residues longer than the corresponding loop in the C-terminal domain and makes numerous inter-barrel contacts. Likewise, loop β4–α4 in the C-terminal domain is extended by six residues, increasing contacts with the N-terminal barrel. The single-domain archaeal Hcy methyltransferases that are homologous to the C-terminal domain are missing these six residues, supporting the notion that this insert evolved to enlarge the inter-domain interface.

### The Zinc Site

The zinc-binding site of MetE is distinguished from most catalytic zinc sites by the presence of four protein ligands to zinc. Mutagenesis of E. coli MetE had previously shown that zinc is bound by a histidine and two cysteines [11] that are equivalent to residues His618, Cys620, and Cys704 in T. maritima MetE. EXAFS studies of the E. coli enzyme indicated a fourth oxygen or nitrogen coordinated to zinc that seemed likely to be a water oxygen. However, the structure of zinc-replete enzyme with folate bound (Table 1) clearly reveals the presence of a fourth protein ligand to zinc. A carboxylate oxygen of the invariant Glu642, which had not previously been identified as a metal ligand, is coordinated to zinc.

In the zinc-replete complexes of MetE with folate that provide resting-state structures of the zinc site, the metal–ligand cluster adopts tetrahedral geometry. EXAFS measurements on substrate-free E. coli MetE are also consistent with tetrahedral coordination [3,11] with bond lengths of 2.31 Å for two Zn–S bonds and 2.04 Å for two nitrogen or oxygen ligands. The observed Zn–S bond lengths in our structure are 2.30 Å, Zn–N is 2.07 Å, and Zn–O is 2.14 Å, in good agreement with the EXAFS measurements.

**Figure 3:**
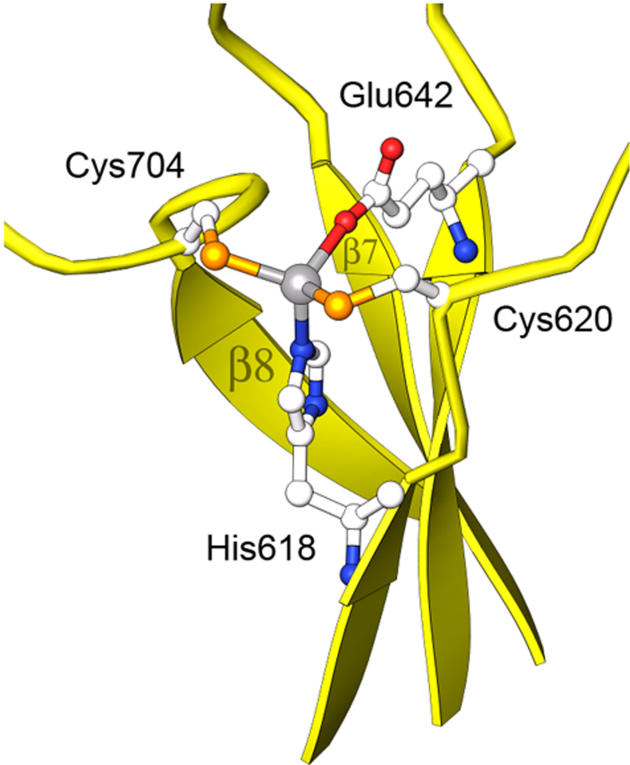
The Resting-State Zinc Is Coordinated in a Tetrahedral Fashion by Four Protein Residues

The residues that bind zinc are located near the ends of barrel strands 5, 6, and 8 (Figure 3). His618 is the C-terminal residue of β5 and Cys620 is located on the following β–α loop. Glu642 is at the C-terminus of β6, and Cys704 resides on the loop following strand β8. The β–α loops that contain the two cysteine residues are drawn together to form the metal-binding site, distorting the barrel (see Discussion).

### The Binary Complex with Hcy

**Figure 4:**
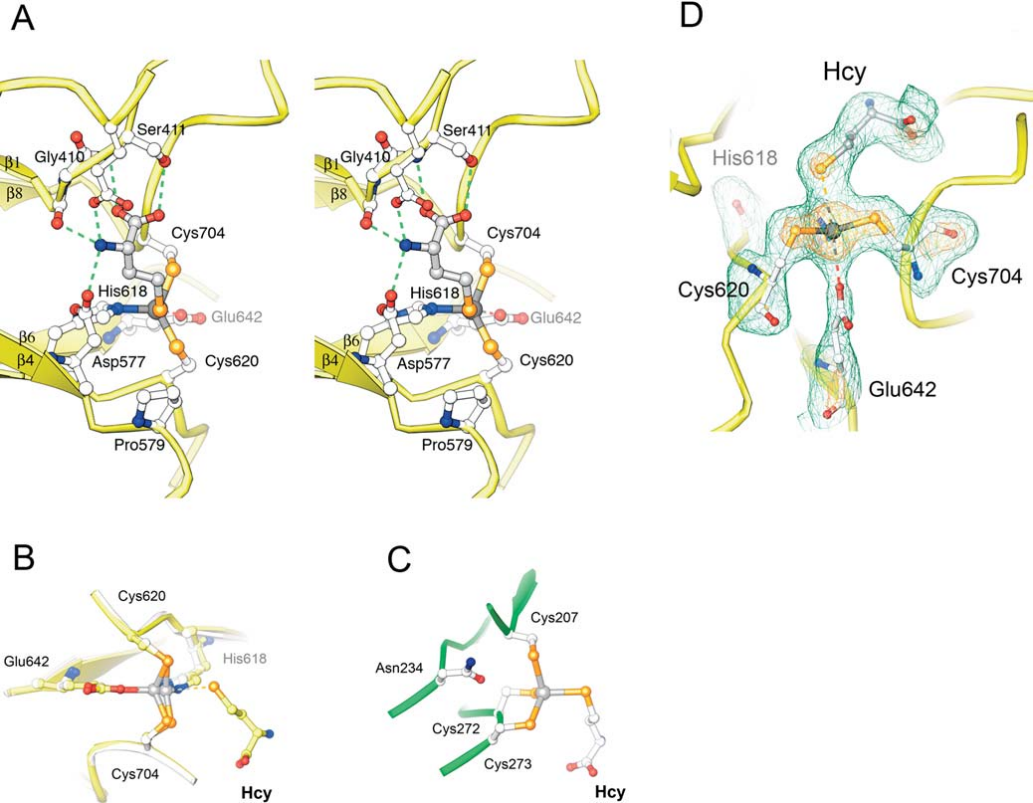
The Geometry at the Zinc Ion in Complexes with Hcy (A) Interactions of Hcy in the MetE·Hcy binary complex. The amino group of Hcy is bound by hydrogen bonds to Asp577, Glu462, and the backbone carbonyl of Ile409; the Hcy carboxyl group interacts with the backbone amide and side chain hydroxyl of Ser411. The Hcy sulfur is coordinated to zinc via a long (3.15 Å) bond, which is eclipsed in this view. (B) Superposition of the MetE resting state (gray) and the Hcy binary complex (yellow). Upon Hcy binding, zinc and His618 move away from Glu642 and closer to Hcy, and the zinc site adopts trigonal bipyramidal geometry with three strong equatorial ligands (Zn–N_His618_, 2.07 Å; Zn–S_Cys620_, 2.23 Å; Zn–S_Cys704_, 2.24 Å) and two distant axial ligands (Zn–O_Glu642_, 2.90 Å; Zn–S_Hcy,_ 3.13 Å). Zinc moves 0.75 Å toward the substrate in the Hcy complex. Full inversion at zinc, upon tight binding of Hcy to MetE, would displace the metal ion approximately 1.5 Å. (C) The MetH·Hcy complex. The zinc configuration in substrate-free MetH is opposite to that found in MetE; binding of Hcy occurs without inversion in MetH and in BHMT. (D) Difference electron density for the MetE·Hcy complex, showing the geometry at the metal-binding site. The map was computed after simulating annealing and refinement of a model omitting the zinc and its five neighbors. The long Zn–Hcy and Zn–Glu642 interactions are indicated with dashes. Contour levels are 2σ (green) and 6σ (orange).

In the complex of Hcy with zinc-replete MetE (Table 1), Hcy is positioned by numerous interactions with conserved protein residues (Figure 4). The amino group is coordinated by hydrogen bonds to Asp577, Glu462, and the carbonyl of Ile409. The carboxyl group of Hcy is bound by the backbone amide and the side chain hydroxyl of Ser411, and hydrophobic contact to the Hcy sulfur is provided by Met468. These interactions with the Hcy substrate are reminiscent of those observed in MetH [2] (see Discussion).

Hcy binding induces significant changes at the metal site (Figure 4). Binding of the substrate sulfur to zinc does not proceed by a simple dissociative reaction in which sulfur is substituted for the oxygen of Glu642. Instead, Hcy approaches the metal ion from the side opposite the Glu642 ligand. This mode of association is unusual; in most catalytic zinc sites the incoming substrate replaces a dissociable fourth ligand without inversion [13]. In the structure of the binary complex determined at pH 5.2, displacement of Glu642 and inversion at Zn^+2^ are incomplete. Zinc coordination changes from tetrahedral to distorted trigonal bipyramidal geometry (Figure 4), and the substrate sulfur and glutamate oxygen both exhibit unusually long ligand–metal distances: 3.15 Å and 2.9 Å for Zn–S and Zn–O, respectively. Comparison with the zinc-replete complex with folate (Figure 4B) shows that zinc and His618 have moved 0.76 Å and 0.98 Å toward the substrate while Cys620, Glu642, and Cys704 remain essentially fixed. The Zn–S(Cys) and Zn–N distances are not significantly altered, but the zinc and these three ligands become more nearly coplanar (Figure 4B). Complete inversion would result in geometry resembling the Hcy complex of MetH (Figure 4C).

The geometry at the metal site in the Hcy complex has been confirmed by omit refinement and by tests with restrained models. The density around the zinc atom in omit maps is well resolved from Hcy but continuous with that of the cysteine ligands (Figure 4D), consistent with a Zn–Hcy distance that is significantly longer than the Zn–Cys bond distances. Imposing tetrahedral restraints in refinements with Hcy as the fourth ligand results in large difference Fourier peaks that also indicate a long Zn–Hcy distance.

Cysteinyl tRNA-synthetase incorporates an active-site zinc with the same set of ligands as MetE and provides a precedent for an inversion at the metal center induced by substrate binding. Cysteinyl tRNA-synthetase uses this zinc ion not for catalysis, but to discriminate against serine, exploiting the strong zinc-thiolate interaction with its substrate [14,15]. In the absence of substrate the zinc site displays geometry that is intermediate between tetrahedral and trigonal bipyramidal. As in MetE, the cysteine substrate binds opposite a glutamate residue and does not displace the glutamate ligand directly. Upon cysteine binding, zinc moves away from glutamate and forms a 2.5 Å bond to cysteine.

### Folate Binary Complex

**Figure 5:**
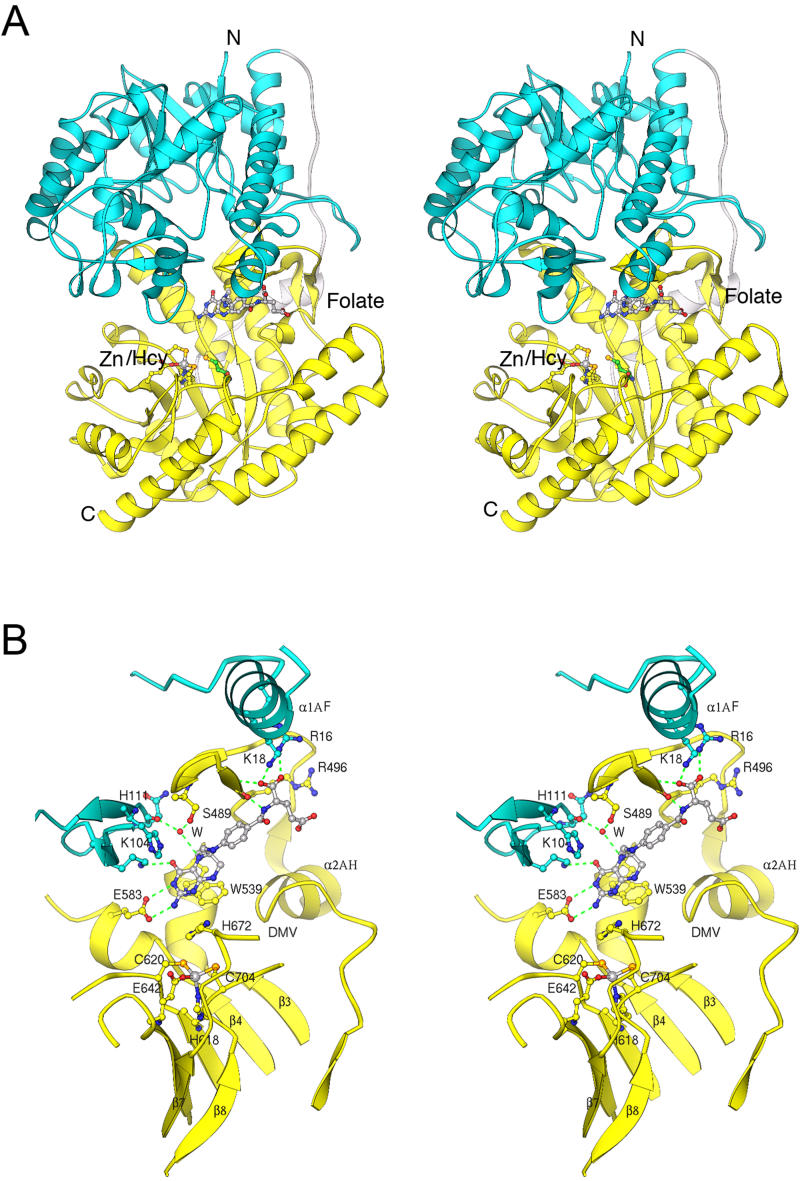
Interactions of CH_3_-H_4_folate with MetE (A) A stereoview of T. maritima MetE showing the substrate and metal-binding sites. This is a composite picture in which Hcy from the MetE·Hcy complex has been positioned by superposition on the structure of the MetE·CH_3_-H_4_folate binary complex. The substrates and metal ligands are displayed in ball-and-stick mode; Hcy is in green. (B) A stereoview of the zinc site and bound CH_3_-H_4_folate. Folate is bound by conserved residues in the N-terminal barrel (aqua) and the C-terminal barrel (yellow) with the N5-CH_3_ facing away from the zinc, at a distance of almost 14 Å. The pterin interacts with the long hairpins of both barrels and with the extra-barrel helices α3AH and α4AH. The groove that binds the glutamate tail of the substrate is bordered by α1AF, by the β hairpin (β2BH and β2CH) and α1AH of the C-terminal domain, and by the conserved DMV sequence that begins α2AH.

Structures of CH_3_-H_4_folate bound to MetE from T. maritima have been determined in both the reduced (zinc-replete) enzyme and the oxidized (disulfide-bonded) form (Table 1). CH_3_-H_4_folate is bound in identical fashion in both structures. The novel feature of these structures is that the MetE·folate complex fails to comply with the classic picture of substrate binding in a TIM barrel. The folate substrate binds in a deep cleft between the two barrels, with its glutamate tail accommodated by a groove in the enzyme surface (Figure 5). The pterin is displaced from the top of the N-terminal barrel, and simultaneously shifted away from the axis of the C-terminal barrel that binds zinc and Hcy (see Figure 2A). It is thus inappropriate to call the N-terminal barrel the “folate-binding domain.” An animation, in which Figure 5 is rotated about its vertical axis, provides a more complete view of the structure and its bound ligands (Video S1).

The pterin ring of CH_3_-H_4_folate is positioned by stacking and by hydrogen bonding with conserved residues (Figure 5). Glu583 makes a bidentate interaction with the 2-NH_2_ and N3 groups of CH_3_-H_4_folate, an arrangement found in several other folate-binding sites. Interaction of N3 with an acidic group has been shown to be important for catalytic activity in dihydrofolate reductase [16], thymidylate synthase [17], and MetH [18]. In the binary complexes with MetE that we report here, the pterin ring of the folate is stacked against Trp539, Lys104 hydrogen bonds to O4, and the folate N5 is hydrogen bonded to a water molecule. However the N8 and N1 positions of the pterin are exposed to solvent.

The subsite that binds the glutamate tail is a groove lined by conserved basic residues (Figure 5). Arg15, Lys18, Arg493, and Arg496 interact with the first glutamyl residue, which is the only one of the three γ-linked glutamate residues that is ordered in the binary complex. Weak binding of the other tail residues is surprising for an enzyme that exhibits an absolute requirement for polyglutamylated folate, but glutamate tails have displayed disorder in other structures where they also contribute to strength of binding [19].

The orientation of bound folate in the binary complexes does not allow transfer of the methyl group to Hcy. As can be seen in Figure 5, the N5-methyl carbon faces away from zinc and Hcy; it is 11 Å from the sulfur of Hcy when the binary complexes are superimposed. A rotation about the folate N10–C4′ dihedral angle, with the interactions of the *para*-amino benzoyl moiety and glutamate acting to anchor the substrate, would position the methyl group correctly with respect to Hcy, decreasing the distance between groups that react to 6.0 Å. This distance is still long, and additional protein or substrate rearrangements would be necessary to close the gap between the sulfur of Hcy and the methyl carbon. An alternative route to a ternary complex that supports methyl transfer would be complete dissociation and reassociation of the CH_3_-H_4_folate. However, because so many interactions with conserved residues are observed in this binary complex, it seems likely to represent an initial intermediate rather than a dead-end complex (see Discussion).

### The ^467^Asp-Met-Val Sequence Mediates Interaction between the Substrate-Binding Sites

**Figure 6:**
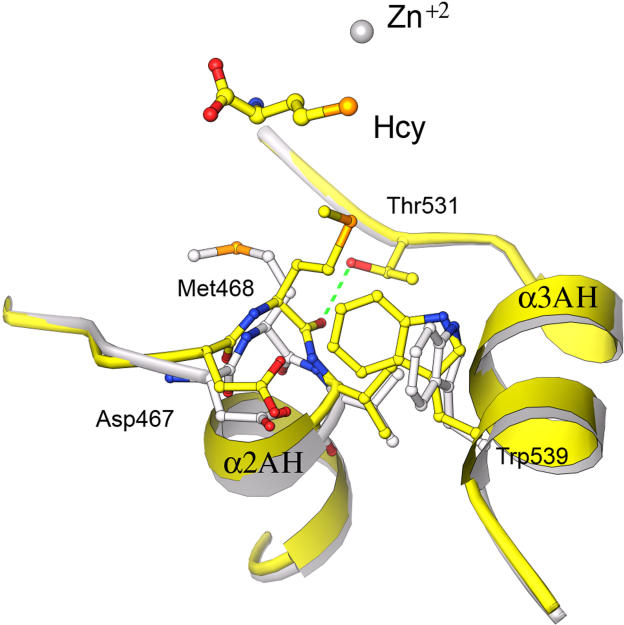
Superposition of the Hcy Binary Complex (Yellow) and Substrate-Free (Gray) Enzymes Showing Local Changes in the DMV Region Met468 moves toward the Hcy substrate, rearranging the start of helix α2AH, and a new hydrogen bond is formed between the carbonyls of Met468 and Thr531. This position of Met468 stabilizes a rotamer of Trp539 that favors folate binding.

One of the fascinating features of the binary substrate complexes is the evidence for communication between the substrate-binding sites, mediated by the invariant ^467^Asp-Met-Val (DMV) sequence, which forms the N-terminal turn of helix α2AH. When Hcy binds, the side chain of Met468 alters its position in concert with backbone displacements of the aspartic acid and methionine residues that start the helix. Comparison of the substrate-free structure with the Hcy binary complex shows how the DMV region moves toward the zinc center when Hcy binds (Figure 6). These changes in turn affect the interactions and orientation of Trp539, favoring the conformation in which Trp539 can stack against the pterin ring. In the absence of substrates, Trp539 can adopt another conformation that would overlap the binding site for the pterin ring.

In the binary complex with CH_3_-H_4_folate, the DMV loop is also recruited to the position it occupies when Hcy is bound. The changes that are induced by binding of folate are reproduced in the complex of folate with the oxidized enzyme. Thus, binding of either substrate favors a conformation that would be expected to increase the affinity of MetE for the other substrate. These small but significant conformation changes observed in the binary complexes precede larger rearrangements that must be induced by binding of both substrates to form a competent ternary complex. Cooperativity in substrate binding could increase the concentrations of the ternary complex and thereby increase turnover in a system that is already plagued by slow chemistry [4].

## Discussion

### Comparisons with MetE from Arabidopsis thaliana

A very recent paper, which appeared after the submission of our manuscript, has described the structure of MetE from A. thaliana [20]. Although the folds of the enzymes from A. thaliana and T. maritima are obviously similar, there are some significant differences in the reported features of the zinc-binding sites. In the complexes of A. thaliana MetE with Hcy or methionine, the distances between zinc and substrate or product sulfur are long, as is the case in the Hcy complex of MetE from *T. maritima,* but water rather than glutamate has been assigned as the ligand opposite to Hcy or Met. In contrast, the electron density in omit maps of the Hcy complex of T. maritima MetE shows no evidence for a water intervening between glutamate and zinc (see Figure 4D). In substrate-free A. thaliana MetE, the metal–ligand bonds are all very long and the geometry is highly distorted, suggesting some disordering or partial oxidation of the metal site under the conditions used for crystallization.

To study folate binding, PteGlu_5_ and CH_3_-H_4_PteGlu_5_ were added to crystals of the Hcy or methionine complexes of A. thaliana MetE [20]. In the resulting structures, the pterin ring is flipped relative to its position in the binary folate complex of T. maritima MetE, and adopts an orientation that is similar to what we anticipated from model building. Although the occupancy of the reduced folate appears to be low, it is estimated that the methyl group of the CH_3_-H_4_PteGlu_5_ is about 7 Å from the sulfur of Hcy, too distant for transfer to Hcy. Thus, both structure analyses suggest that additional conformation changes must occur to form a reactive ternary complex.

### Comparisons of MetE with MetH

MetE and MetH display no detectable sequence homology and have different sets of zinc ligands. Comparison of the barrels from MetE with the corresponding domains of MetH that bind folate or Hcy reveal that the N-terminal barrel of MetE, which carries some folate-binding determinants, differs in significant ways from the folate barrel of MetH, whereas the Hcy barrels share many similar features.

Two other (βα)_8_ barrels that bind CH_3_-H_4_folate have been described: methyltetrahydrofolate corrinoid/iron-sulfur protein methyltransferase [21] and the folate-binding module of MetH [2]. These homologous barrels both bind the CH_3_-H_4_folate substrate at the top of the folate barrel and use similar interactions with residues contributed by the C-termini of the inner barrel strands. In MetE, CH_3_-H_4_folate is displaced from the N-terminal barrel and bound primarily by residues in the long extra-barrel β hairpin of the C-terminal Hcy domain (see Figures 2A and 5). Dissimilarities of the decorating loops in the N-terminal barrel of MetE and the folate barrel of MetH are documented by poor statistics for sequence matches and for alignments with the structures of corrinoid/iron-sulfur protein methyltransferase or MetH.

**Figure 7:**
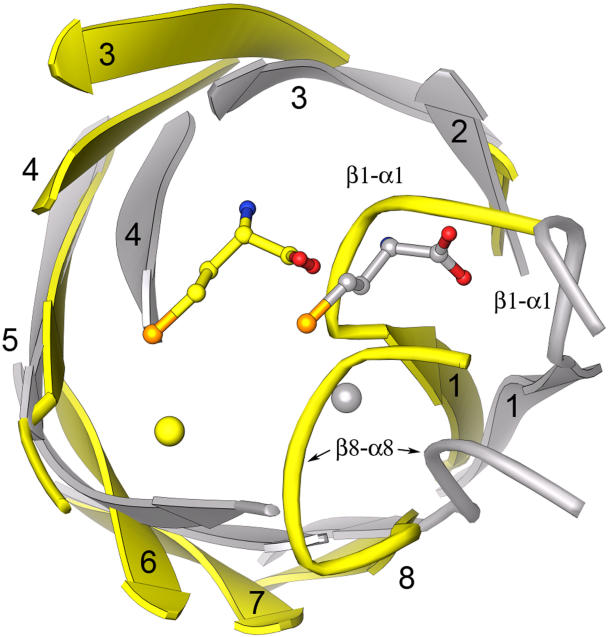
Superposition of MetE and MetH Zn^+2^/Hcy Barrels In MetH (gray) the sulfur of Hcy is positioned close to the center of the barrel for interaction with methylcobalamin. In MetE (yellow) the α1–β1 and α8–β8 connectors make large incursions across the top of the barrel, displacing Hcy to the other side of the barrel.

The Hcy barrels of MetE and MetH are compared in Figure 7, which shows how each structure accommodates metal binding. Strand distortions in the Hcy barrels, which have been associated with construction of a metal-binding site [2,9], are related but not identical in MetE and MetH (or BHMT [9]). In MetH, strand β7 is extruded from the barrel and strands β6 and β8 are pinched together to bind the zinc [2]. In MetE, strands β6 and β7 are displaced relative to their positions in MetH, allowing strands β5 and β8 to approach one another. In both enzymes, distortion of β8 is accompanied by a splay in strand β1; only one classic hydrogen bond is made between strands β1 and β2. Conserved residues in MetH, MetE, and BHMT stabilize inter-strand interactions by forming side-chain-to-main-chain hydrogen bonds.

Major differences in the connecting loops β1–α1 and β8–α8 displace the zinc and Hcy sites in MetE relative to MetH by approximately 6 Å so that the sulfur of Hcy is no longer on the barrel axis but is shifted toward one wall of the barrel (Figure 7). The altered positions of the ligands and the binding of zinc by Glu642 lead to inversion of the zinc center relative to its configuration in MetH. Displacement of the Zn^+2^/Hcy site and the unusual mode of folate binding seem to have evolved to allow methyl transfer in a ternary E·Hcy·CH_3_-H_4_folate complex.

Despite the translation and inversion of the metal and its ligands, the orientation and local interactions of Hcy are almost identical in MetE and MetH (Figure 7). Both enzymes use conserved carboxylate residues to interact with the amino group of Hcy, forming salt bridges (see Figure 4). In both MetH and BHMT, the carboxyl group of Hcy is bound by a pair of backbone amides located at the beginning of the β1–α1 extension. In MetE the corresponding interactions of COO^−^ are made by the backbone amide and the side chain hydroxyl of Ser411, again located on the extension following β1.

Curiously, it appears that distortions of the barrel strands and connecting loops need not be undone when metal binding is lost through evolution. Distortions of the barrel strands and their downstream loops are retained in the N-terminal barrel of MetE despite loss of metal and Hcy binding, and similar distortions were first observed in uroporphyrinogen decarboxylase [22], which is also not a metalloenzyme. Despite the lack of detectable sequence homology, uroporphyrinogen decarboxylase is the closest structural relative of the Hcy domain of MetE in the current protein database: alignment using DALI [23] matches the two folds with a similarity score that is higher than that for the Hcy barrels from MetE and MetH. Uroporphyrinogen decarboxylase may have evolved from a Zn^+2^/Hcy barrel, despite the fact that it is no longer a zinc-dependent thiol alkyltransferase.

### Substrate Binding and Activation: Inferences from the Structures

In the structure of the MetE·Hcy complex, the zinc site adopts distorted trigonal bipyramidal geometry with long bonds from the metal ion to glutamate and Hcy (see Figure 4). In contrast, EXAFS measurements on the Se–Hcy complex of E. coli MetE at pH 7.2 are best fit to a tetrahedral ligand environment with two sulfurs (2.33 Å), one nitrogen or oxygen (2.02 Å), and one selenium (2.433 Å), in which the longer Zn–Se distance reflects the increased radius of selenium relative to sulfur [3]. EXAFS studies of the related methyltransferase MT2-A, which has the same set of zinc ligands as MetE, also indicate tetrahedral geometry in the binary complex with coenzyme M (2-mercaptoethanesulfonic acid) [7]. Both of these studies concluded that the geometry at zinc does not change appreciably upon binding of the thiolate substrate.

Because the crystals of the Hcy binary complex of MetE were equilibrated at pH 5.2, it is possible that Hcy may be protonated in the X-ray structure. Thus, the long S–Zn bond and partial inversion at zinc might be explained by the relatively weak interaction between the thiol and the metal ion, as documented for thiol and thioether ligands in model compounds [24,25,26]. X-ray studies of Hcy binding at neutral pH and EXAFS measurements at lower pH will be necessary to determine whether pH is a key parameter that affects the metal–ligand geometry. It is possible that complete inversion of zinc geometry will be observed in the structure of the MetE·Hcy complex at neutral pH.

A long Zn–S^−^ bond may be functionally important. It has been suggested that a long bond and/or distorted geometry would optimize the reactivity of zinc-dependent thiol alkyltranferases [27] by increasing the charge on the thiolate sulfur, and would avoid trapping of a lower-energy tetrahedral species. Since zinc is known to have flexible coordination geometry [28], a five-coordinate state seems plausible, and the observed structure of MetE·Hcy may indeed afford a glimpse of an intermediate or transition-like state that is poised to attack the N5 methyl group of the folate substrate.

The motion of Zn^+2^ that accompanies Hcy binding to MetE is unique among the Hcy methyltransferases with known structures. There is no evidence for inversion of configuration at zinc in MetH, and inversion is precluded in BHMT, where a leucine occupies the position analogous Glu642 of MetE. Zinc motion provides a novel way to alter the distribution of charge among the zinc and its ligands, thereby modulating thiolate reactivity. The effects on the electronic structure could be larger than those resulting from the changes in bond lengths observed in other zinc-dependent alkyltransferases [29,30]. By analogy with a proposal for the reaction cycle of protein farnesyltransferase [30], reassociation of Glu642 could promote dissociation of the methionine product following methyl transfer.

An unexpected feature of the MetE structures is the binding mode of CH_3_-H_4_folate, with the pterin ring incorrectly oriented for methyl transfer. It is possible that folate binds initially in this manner to avoid blocking access to the Hcy binding site, which lies between zinc and CH_3_-H_4_folate. Space-filling models show that the Hcy-binding site remains accessible in the MetE·CH_3_-H_4_folate binary complex. The observed binding mode thus permits random addition of substrates but does not rule out a kinetically preferred order of binding.

In both MetH and MetE, protonation of methyltetrahydrofolate is thought to occur in the ternary complexes but not in the binary folate complexes [4]. The immediate proton donor has not yet been identified by enzymatic or biochemical studies of E. coli MetE. Formation of the binary Hcy complex results in release of a proton to solvent (Z. S. Zhou and R. G. Matthews, unpublished data). Thus Hcy is not a likely proton donor. It is more likely that an active-site residue may serve as an acid catalyst. Structures of the binary complexes do not implicate a particular residue as a general acid catalyst, but suggest possible candidates. His111 could serve as a proton donor through water if protonation were to occur before folate rearrangement. His672, part of a conserved ^670^Asp-Ile-His-Ser-Pro sequence, or Asp467, located in the DMV loop, may be positioned to serve as donors if protonation occurs after folate rearrangement. All three of these candidate residues are invariant in multiple sequence alignments.

### Gene Duplication of a Sequence Encoding the Hcy Barrel

Earlier comparisons of sequences had suggested that MetE evolved by gene duplication. The two domains of E. coli MetE share a conserved Gln-Ile-Asp-Glu-Pro-Ala repeat [31], and the sequences display 39% identity (50% similarity) within the regions now seen to span β3 to α4A of the two barrels. The pseudosymmetry of the structural features decorating the barrels provides compelling evidence for a close relationship between the two halves of MetE, verifying the inferences based on sequence alignments. The cores of (βα)_8_ barrels, though they display characteristic distortions and can be classified into subgroups [32], do not provide as strong evidence for relatedness as do the similarities of regions inserted in intervening loops. It is difficult to ascertain whether the regions responsible for folate binding were inserted before or after gene duplication. The long β2–α2 loop in the N-terminal barrel and corresponding sequences in archaeal relatives of MetE favor the notion that precursors of folate-binding segments were present before duplication. In any case, folate-binding determinants have developed or been retained primarily in the C-terminal Hcy barrel but not in its N-terminal replicate.

The idea that the zinc/Hcy barrel is the ancestral fold is based on several lines of evidence. This barrel shows significant structural homology to a broad family of zinc-dependent thiol methyltransferases, including not only MetH and BHMT but also the single-domain archaeal transferase enzymes that react with methylcobalamin. Indeed it seems likely that the three enzymes that convert Hcy to methionine, MetE, MetH, and BHMT, are all descended from a primordial zinc/Hcy barrel. In contrast, DALI searches that assess structural similarities [23] reveal that the N-terminal barrel is more similar to the C-terminal barrel of MetE than to any other known protein, suggesting that its immediate precursor is the Hcy barrel of MetE.

**Figure 8:**
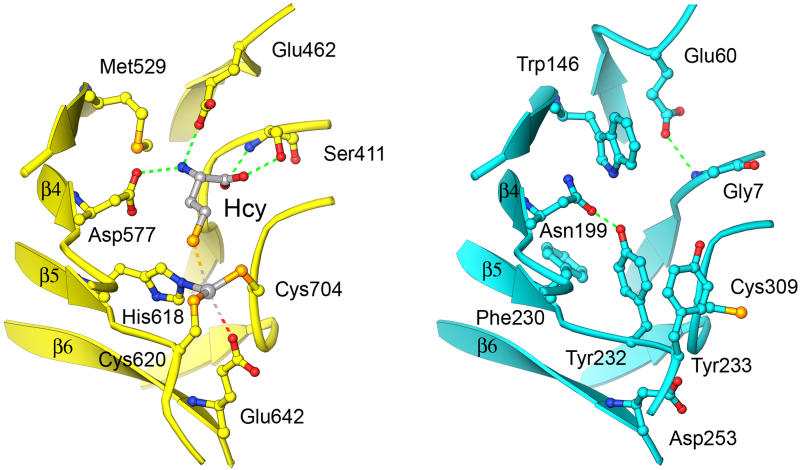
Disruption of Hcy and Zinc Binding in the N-Terminal Barrel Overhaul of the Zn^+2^/Hcy site in the N-terminal domain (aqua) following gene duplication is accomplished through mutation and small backbone displacements. The competent Zn/Hcy site from the C-terminal barrel is at the left; the remodeled site from the N-terminal barrel is at the right. Important substitutions that disable Hcy and zinc binding are Trp146, Phe230, Tyr232, and Asp253 (see text).

Gene duplication of a Zn^+2^/Hcy barrel would have replicated the sites that bind Zn^+2^ and Hcy, but these sites have been disabled in the N-terminal barrel of contemporary MetE. Disruption of zinc and Hcy binding is effected by both residue mutations and backbone conformational changes (Figure 8). Mutation of the equivalent of Cys620 in the Hcy barrel to Tyr232 leads to stacking with the adjacent Tyr233 (phenylalanine in most MetE sequences) and results in a major backbone conformational change. The hydroxyl group of the Tyr232 forms a hydrogen bond to Asn199 of the conserved Leu-Val-Asn-Glu-Pro-Ala sequence at the β4–α4 loop and thus removes a crucial Hcy-binding determinant. Although Cys309, the equivalent of Cys704 of the C-terminal barrel, is retained in most MetE sequences, mutation of the other zinc ligands and many of the Hcy-binding residues leads to a complete overhaul of the binding site for Hcy.

### Why Is a Second Barrel Recruited for a Ternary Complex Mechanism?

Although an entire barrel domain is recruited to elicit direct methyl transfer from folate to Hcy, the structure reveals that the functional groups of this domain are mostly disabled. The binding determinants and potential catalytic residues are located primarily in extensions and excursions of the C-terminal Zn^+2^/Hcy barrel rather than in the “new” N-terminal domain. Duplication and divergence of an entire barrel is an elaborate strategy to accommodate a second relatively large substrate at a single active site, and MetE provides the first instance of the face-to-face barrel construction that is required to build such an active site. A more common strategy to accommodate a ternary complex is exemplified by the related Hcy methyltransferase, BHMT. In this enzyme the site for the rather small second substrate, betaine, is constructed in part from β–α barrel extensions and in part from a dimerization arm that is appended to the barrel and contributes to the active site of the partner chain of the functional dimer.

The N-terminal domain of MetE may nevertheless be essential for ternary complex formation and catalysis. In the family of thiol alkyltransferases, quenching of opposite charges on Hcy and the alkyl donor is believed to drive the reactions, and a hydrophobic environment [33] and desolvation [34] of reactants may be critical for reactivity. Rearrangement of folate to form a viable ternary complex may be accompanied by rearrangement or closure of domains around the substrate-binding cleft that would reposition key residues and isolate the active site from solvent. Hcy binding is accompanied by a contraction of the top of the C-terminal barrel that alters the relative positions of the N- and C-terminal barrels. This small domain shift clearly gives hints of inter-domain flexibility. On the other hand, the commissioning of the N-terminal domain may reflect an evolutionary strategy in which gene duplication is utilized as the most facile way to recruit additional sequences. Structures of ternary complexes, obtained using mutant enzymes and/or substrate analogs, should provide further insights into the methyl transfer reaction and the functional roles of the N-terminal domain.

## Materials and Methods

### 

#### Cloning and purification

The TM1286 gene was PCR amplified from T. maritima genomic DNA ( ATCC, Manassas, Virginia, United Stats) and cloned into pET-151D/TOPO (Invitrogen, Carlsbad, California, United States). The expression construct contains an N-terminal leader sequence consisting of a 6X histidine tag followed by a V5 epitope, rTEV cleavage site, and residues 2–734 of the coding sequence of TM1286. This vector was overexpressed in BL21(DE3)Star (Invitrogen) by induction with 0.8 mM IPTG for 8 h in LB media supplemented with zinc sulfate. The histidine-tagged protein was purified by a 10-min 70 °C heat step, which precipitates heat-labile protein, followed by affinity chromatography on Zn(II)-NTA and elution with a 50 mM to 1.5 M glycine gradient. The protein was dialyzed against 50 mM Tris (pH 7.4) and 500 μM Tris(2-carboxylethyl)phosphine (TCEP) and concentrated to 20 mg/ml. Selenomethionine-labeled protein was expressed in M9 medium supplemented with amino acids and zinc sulfate.

#### Crystallization

TM1286 was crystallized by the vapor batch (microbatch) method under oil utilizing 96-well Douglas vapor batch plates and a 1:1 mixture of silicon:paraffin oil (Hampton Research, Alliso Viejo, California, United States). Orthorhombic crystals of space group P2_1_2_1_2 (*a* = 163.57 Å, *b* = 158.76 Å, *c* = 64.16 Å, α = β = γ = 90°) were grown by mixing 20 mg/ml protein 1:1 with 25% poly(ethylene glycol) 4000, 0.2 M ammonium sulfate, and 0.1 M sodium acetate (pH 4.6). Selenomethionine-labeled protein was crystallized by mixing 1:1 with 12% poly(ethylene glycol) 4000, 0.2 M ammonium sulfate, and 0.1 M sodium acetate (pH 4.6). Anapoe detergents (Anatrace, Maumee, Ohio, United States) were used as additives and seen to have a favorable effect on crystal morphology. Crystals in a cryoprotectant solution consisting of 15% poly(ethylene glycol) 4000, 0.12 M ammonium sulfate, 0.1 M sodium acetate (pH 5.2), and 12.6%–14.1% meso-erythritol were flash-cooled in liquid nitrogen.

These crystals were found to be depleted of zinc with a disulfide bond connecting the zinc ligands, Cys620 and Cys704. Zinc-replete crystals were obtained by soaking in cryoprotectant solution containing 500 μM zinc sulfate and 500 μM TCEP for 4–16 h prior to flash cooling. The enzyme:folate binary complex was formed by soaking crystals in cryoprotective solution with added 3.5 mM CH_3_-H_4_PteGlu_3_ (a gift from Rebecca E. Taurog) for several hours. The enzyme:Hcy binary complex was formed by soaking crystals pre-equilibrated with zinc sulfate and TCEP in cryoprotective solution containing 10 mM L-Hcy (a gift from Rebecca E. Taurog) for several hours.

#### Phasing and refinement

All datasets were collected at the Advanced Photon Source (APS) at Argonne National Laboratory. Data collected at the DND-CAT beamline on a Mar225 detector were processed with XDS [35] whereas those collected at COM-CAT on a Mar165 detector were processed with DENZO/SCALEPACK [36]. Statistics for the datasets appear in Table 1.

Experimental phases to 2.8 Å were derived from selenium SAD measurements at the selenium peak using heavy atom sites located by a three-wavelength selenium MAD experiment at lower resolution. Thirty of the 36 expected selenium sites were found and phases were determined using SOLVE version 2.05 [37], and statistical density modification was performed in RESOLVE [38]. The initial model from RESOLVE was rebuilt in MI-fit [39] and partly refined, and was used with the molecular replacement program EPMR [40] to determine the higher resolution structure of oxidized MetE at 2.00 Å. A search model derived from the refined oxidized structure was subsequently used to solve the substrate complexes with EPMR. All models were developed by refitting and rebuilding in MI-fit alternated with refinement in CNS version 1.1 [41]. Refinement protocols included simulated annealing with torsional dynamics, coordinate minimization, and adjustment of individual B-factors.

In late rounds of refinement of the binary complexes, weak restraints were applied to maintain the geometry at the zinc site. For the MetE·CH_3_-H_4_folate complexes (the resting state of the zinc center), restraints were based on ideal tetrahedral geometry; for the Hcy complex, restraints were chosen using bond valence sums analysis [42] to make the bond lengths compatible with the known +2 oxidation state of zinc. The resting-state zinc site (Zn–N_His618_, 2.07 Å; Zn–S_Cys620_, 2.31 Å; Zn–S_Cys704_, 2.29 Å; Zn–O_Glu642_, 2.14 Å) gives a bond valence sum of 1.88, consistent with the known +2 oxidation state of zinc, and a net contraction of Zn–S_Cys_ bonds from 2.30 to 2.24 Å upon Hcy binding is enough to maintain the bond valence sum for the five-coordinate state with long axial bonds.

## Supporting Information

Coordinates of the structures have been deposited in the Research Collaboratory for Structural Bioinformatics' Protein Data Bank (http://www.rcsb.org/pdb/) with accession codes 1T7L (substrate-free oxidized), 1XDJ (zinc and Hcy complex), 1XPG (zinc and methyltetrahydrofolate complex), and 1XR2 (oxidized methyltetrahydrofolate complex).

Video S1 Video of MetE Showing the Substrate and Metal-Binding Sites (7.9 MB WMV).

### Accession Numbers

The SwissProt (http://www.ebi.ac.uk/swissprot/) accession numbers for the gene products discussed in this paper are A. thaliana MetE (SwissProt O50008), E. coli MetE (SwissProt P25665), S. cerevisiae MetE (SwissProt P05694), T. maritima MetE/TM1286 (SwissProt Q9X112), and MetH (SwissProt P13009).

## Abbreviations

APS: Advanced Photon Source
BHMT: betaine–homocysteine methyltransferase
DMV: ^467^Asp-Met-Val
EXAFS: extended X-ray absorption fine structure
Hcy: L-homocysteine
MetE: cobalamin-independent methionine synthase
MetH: cobalamin-dependent methionine synthase
CH_3_-H_4_folate: N5-methyl-5,6,7,8-tetrahydrofolate
CH_3_-H_4_PteGlu_3_: N5-methyl-5,6,7,8-tetrahydropteroyl-(tri)-γ-L-glutamate
TIM: triose phosphate isomerase
TCEP: Tris(2-carboxylethyl)phosphine
